# Monitoring of circulating tumour-associated DNA as a prognostic tool for oral squamous cell carcinoma

**DOI:** 10.1038/sj.bjc.6602801

**Published:** 2005-10-04

**Authors:** E Thomas, R J Shaw, J M Risk

**Affiliations:** 1 Molecular Genetics and Oncology Group, School of Dental Sciences, The University of Liverpool, Liverpool, UK; 2 Regional Maxillofacial Unit, University Hospital Aintree, Liverpool, UK

**Sir**,

We note the recent paper of Hamana *et al* (2005) in your journal with interest and have some observations in the light of a similar pilot study that we presented last year at the 2004 International Conference on Head & Neck Cancer (Thomas *et al*, 2004).

Our study aimed to establish whether genetic alterations observed in preoperative circulating DNA were maintained after surgical treatment and if this could be related to outcome. Similarly to the Hamana study, DNA was isolated from tumour tissue, white blood cells as control DNA, pre- and postoperative plasma from each of six oral cancer patients and microsatellite analysis was used to measure allelic imbalance (AI). However, in our study 22 markers were used instead of nine, with the result that AI was detected in 100% of tumours (six of six) rather than the 59% (38 of 64) observed in the Hamana study. Our markers were also chosen to include those that have previously demonstrated AI in oral SCC and we were surprised to note that Hamana *et al* did not include any markers from 3p21, 3p14 or 17p13.1. The other main difference in our study was that our clinical follow-up and investigation of plasma specimens continued for a minimum of 2 years.

In four of six (67%) of our patients, presurgery circulating DNA showed allelic imbalance at >1 locus that demonstrated AI in tumour DNA. This is similar to the data obtained by Hamana *et al*, who observed AI in preoperative plasma in 28 of their 38 (74%) patients with AI-positive tumours. Although we have no data *immediately* postsurgery, it was interesting to note that the incidence of AI in circulating DNA in the Hamana study DECREASED (13 of 38; 34%) at this time point, when it would be expected to remain high due to tumour-specific DNA persisting in the circulation before degradation and clearance. However, the authors do not indicate the exact time of sampling for their postsurgery plasma and these data may be a result of reduced sensitivity of the AI technique when nontumour DNA is released into the bloodstream during the trauma of surgery. Our postoperative follow-up plasma samples were obtained at 24–48 months and AI was retained at five of the nine and one of the eight markers in samples from two patients who developed locoregional recurrence. None of our patients developed distant metastases. Our data show the potential for this technique in the early detection of locoregional recurrence and augments the data of Hamana *et al*, which shows value in predicting distant metastases. Indeed their data suggest that persistence of DNA aberrations at 4 weeks after surgery had a 100% positive predictive value for distant metastases. Four weeks is often the point used to prescribe postoperative radiotherapy or combined chemo-radiotherapy (POCRT). In the context of recent trials advocating POCRT in poor prognosis head and neck cancer (Cooper *et al*, 2004; Bernier *et al*, 2005), this finding offers promise in clinical translation.

The potential for use of circulating DNA in head and neck cancer has been demonstrated by this and other studies (Nawroz-Danish *et al*, 2004); however, the question arises as to the best method of analysing genetic or epigenetic aberrations. Most patients are noninformative for at least one marker and these AI techniques become insensitive in the face of ‘contamination’ with normal DNA such as after trauma, surgery or blood transfusion. We speculate that other more sensitive techniques may be more suited to this rapidly developing field, and clearly, further high-quality studies such as that of Hamana *et al* are required.

## References

[bib1] Bernier J, Pfister DG, Cooper JS (2005) Adjuvant chemo- and radiotherapy for poor prognosis head and neck squamous cell carcinomas. Crit Rev Oncol Hematol (in press) 10.1016/j.critrevonc.2005.04.010 15979887

[bib2] Cooper JS, Pajak TF, Forastiere AA, Jacobs J, Campbell BH, Saxman SB, Kish JA, Kim HE, Cmelak AJ, Rotman M, Machtay M, Ensley JF, Chao KS, Schultz CJ, Lee N, Fu KK (2004) Postoperative concurrent radiotherapy and chemotherapy for high-risk squamous-cell carcinoma of the head and neck. N Engl J Med 350: 1937 15128893 10.1056/NEJMoa032646

[bib3] Hamana K, Uzawa K, Ogawara K, Shiiba M, Bukawa H, Yokoe H, Tanzawa H (2005) Monitoring of circulating tumour-associated DNA as a prognostic tool for oral squamous cell carcinoma. Br J Cancer 92: 2181 15928666 10.1038/sj.bjc.6602635 PMC2361808

[bib4] Nawroz-Danish H, Eisenberger CF, Yoo GH, Wu L, Koch W, Black C, Ensley JF, Wei WZ, Sidransky D (2004) Microsatellite analysis of serum DNA in patients with head and neck cancer. Int J Cancer 111: 96 15185349 10.1002/ijc.20240

[bib5] Thomas E, Shaw RJ, Sutton DN, Rogers SN, Field JK, Risk JM, Woolgar JA, Field EA (2004) Does longitudinal analysis of circulating DNA in the plasma predict outcome in oral cancer? Sixth International Head & Neck Conference, Washington, USA. 7th August 2004

